# Tandem Walk in Simulated Martian Gravity and Visual Environment

**DOI:** 10.3390/brainsci12101268

**Published:** 2022-09-20

**Authors:** Marissa J. Rosenberg, Matthew Koslovsky, Matthew Noyes, Millard F. Reschke, Gilles Clément

**Affiliations:** 1 KBR, Houston, TX 77058, USA; 2 Department of Statistics, Colorado State University, Fort Collins, CO 80523, USA; 3 Neuroscience Laboratory, NASA Johnson Space Center, Houston, TX 77058, USA

**Keywords:** spatial disorientation, virtual reality, Martian gravity, tandem walk

## Abstract

Astronauts returning from long-duration spaceflights experience visual-vestibular conflicts that causes motion sickness, perceptions that the environment is moving when it is not, and problems with walking and other functional tasks. To evaluate whether astronauts will have similar decrements after they land on Mars following exposure to weightlessness, participants were held by a device that offloads their weight, first entirely (0 G), and then partially (0.38 G) or not at all (1 G). Tandem (heel-to-toe) walk on a medium-density foam surface was used to assess the subject’s walking performance. Two visual conditions in virtual reality were investigated: normal vision and a visual-vestibular conflict generated by disorienting optokinetic stimulation (DOS). Tandem walking performance with DOS was better in 0.38 G compared to 1 G. Tandem walking performance in DOS in 1 G was not significantly different from tandem walking performance after spaceflight or bed rest. The increased tandem walking performance in 0.38 G compared to 1 G was presumably due to an increased cone of stability, allowing a larger amplitude of body sway without resulting in a fall. Tandem walking on a compliant foam surface with a visual-vestibular conflict is a potential analog for simulating postflight dynamic balance deficits in astronauts.

## 1. Introduction

Visual-vestibular alterations occur during critical periods of space travel, such as during entry into weightlessness and during return to Earth’s gravity. Disturbances induced through visual-vestibular conflict typically include motion sickness, perceptual illusions, changes in eye-head coordination, and alteration in balance and gait after landing. The longer astronauts are exposed to microgravity, the more intensely they experience these disturbances. This pattern poses a significant challenge for astronauts who participate in future long-duration exploration missions to the Moon or Mars. The current scenario for a human Mars mission includes an eight-month journey in weightlessness (0 G) before landing. It is therefore critical to assess how humans will perform in Martian gravity after they have adapted to 0 G [1].

During reentry into a gravitational field, astronauts commonly report exaggerated motion of their surroundings when they move their head [2]. While performing head movements in pitch, several crewmembers reported a perceived translation of the visual surroundings and the sensation that they were accelerating faster than they actually were. In addition, their perceived self-translation and perceived head roll were greater in rate and displacement amplitude than the actual head roll. The displacement amplitude of yaw head movements made immediately after landing also felt exaggerated [3].

Returning crewmembers also experience difficulties standing and walking, especially with their eyes closed. They experience postural instability and walk slowly even after missions of relatively short duration, whereas spaceflight-induced changes in their muscular strength are minimal due to the efficacy of in-flight exercise countermeasures [4,5]. Thus, postural changes cannot be solely ascribed to spaceflight-induced skeletal muscle changes. It appears that some of these deficits are due to gaze destabilization (oscillopsia) because of a reduced ability to engage in compensatory head pitch movements during locomotion [6]. The organization of astronauts’ gait pattern is only slightly disrupted after spaceflight, which indicates that they rely significantly on visual feedback when walking after their return from space since their vestibular system is decidedly impaired.

When walking, head stabilization contributes to dynamic balance because head movements in pitch compensate for the vertical trunk translation that occurs during each step of the gait cycle [7]. In addition to head stabilization, the vestibulo-ocular reflex compensates for head movements to stabilize gaze in space. This coordinated strategy between the motion of the eye, head, and trunk plays a central role in maintaining clear vision during natural body movements [4]. The otolith organs of the vestibular system participate in the gain of the vestibulo-ocular reflex during movement of the head in pitch and in yaw. Individuals with vestibular deficits also restrict their head movements during locomotion [8]. On Earth, the otolith signals provide information about head orientation relative to gravity. In weightlessness, they are effectively unloaded, and consequently, the CNS interpret all otolith output signals as due to head translation, not head tilt. This reinterpretation continues for several hours after the return to Earth. Alterations in the control of astronauts’ eye movements, posture, and gait after spaceflight support this hypothesis [9,10,11]. Recent evidence suggests that spaceflight induces a change in neuroplasticity, specifically in the motor and the vestibular cortical regions [12]. Both positive and negative plasticity occur, including reorganization and decrease in volume of the cortical areas associated with behavioral experience. The changes in cortical topographic organization for sensory and motor regions during long-duration spaceflight may reduce vestibular function and motor control abilities during landing on Mars [13]. Interestingly, body unloading and reduced proprioceptive and vestibular input during long-duration (30–70 days) bed rest induce a loss of tonic muscle activation after bed rest and subsequent postural and locomotor instability [14,15,16].

During the Apollo missions, astronauts walked slower during extra-vehicular activities on the lunar surface than they did on Earth [17]. However, falls and near falls were frequent due to the ruggedness of the terrain, limited mobility and visibility in the suit, and astronauts’ automatic postural reactions not correcting the disequilibrium. Indeed, the automatic postural reactions in response to tipping or stumbling occurred too fast in lunar gravity (0.16 G), so these reactions caused further disruption to astronauts’ balance [18].

Studying the visual-vestibular conflict that occurs through adaptation of the vestibular system in weightlessness is difficult in a normal 1 G environment. Thus, researchers have not been able to directly study the capability of humans to walk in Martian gravity (0.38 G). Rather than modifying the gain of the vestibular system, experimental protocols frequently rely on modifying visual input to induce visual-vestibular conflict. Ground-based simulations using suspension devices have been used to predict the mechanical work and metabolic expenditures required for walking and running in Martian gravity [19], but no one has studied the relative contribution of visual and somatosensory cues to dynamic balance in Martian gravity.

The first objective of this study was to investigate how exaggerated motion of visual surroundings affects dynamic balance in simulated Martian gravity. The second objective was to simulate the walking deficits seen in astronauts after spaceflight for evaluating potential countermeasures. A virtual reality environment simulated the astronauts’ postflight illusory movements caused by head movements. A suspension device was used to simulate weightlessness (0 G) and Martian gravity (0.38 G). The tandem walking test was used to assess dynamic balance control in this simulated visual and gravitational environment. We chose the tandem walking test because it is used in clinics to assess patients who may be at risk for falls [20]; it has also been used to evaluate dynamic balance control in astronauts returning from spaceflight [2,14,15,21]. The third objective of this study was to compare the tandem walk performance in simulated weightlessness and Martian gravity in our study with previously collected data from tandem walks on a hard floor with astronauts with closed eyes after spaceflight and in subjects after bed rest [14,15].

## 2. Materials and Methods

### 2.1. Participants

Ten healthy volunteers (five male, five female; aged 32.3 ± 5.3 years; mean ± SD) participated in this study. Subject were selected through NASA’s test subject screening procedures. All subjects passed a Class III flight physical. This study was carried out in accordance with the recommendations of the NASA Johnson Space Center Institutional Review Board and were performed in accordance with the ethical standards established by the 1964 Declaration of Helsinki. All subjects provided written, informed consent before participating in the study. The protocol was approved by the NASA Johnson Space Center Institutional Review Board. All tests were conducted at the NASA Johnson Space Center, Houston, TX, USA.

### 2.2. Active Response Gravity Offload System (ARGOS)

**Figure 1:**
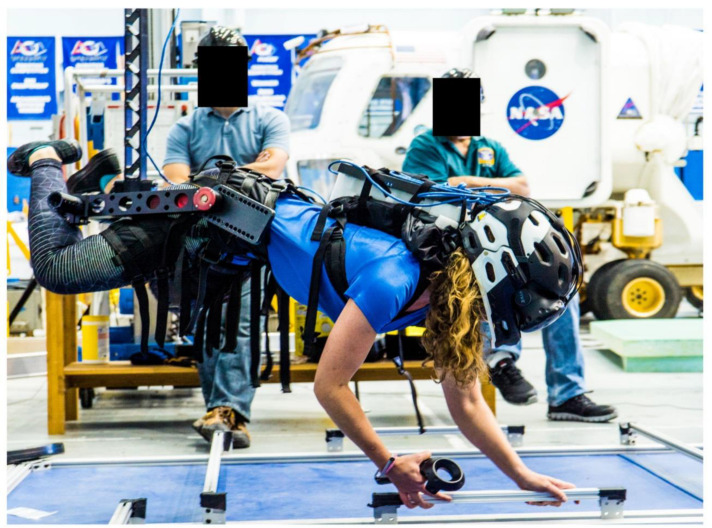
NASA’s Active Response Gravity Offload System (ARGOS) is used to offload 100% of the subject’s body weight (simulated 0 G). The subject is viewing handholds in virtual reality and using physical handholds to move herself around the environment.

The NASA ARGOS system allows human subjects to perform functional tasks in simulated reduced gravity (Figure 1). Subjects can move freely inside a 12.5-m long × 7.3-m wide × 7.3-m high steel structure while in a harness suspended by a steel cable. ARGOS uses robotic mechanisms and computer-controlled electric motors to supply a continuous vertical offload of 0% (1 G) to 100% (0 G) of a person’s weight during walking, running, and jumping. Rotational motion is also accommodated by various gimbal interface mechanisms [22]. For this study, ARGOS was utilized to off-load 62% of a person’s weight to enable testing in a 0.38 G setting.

### 2.3. Virtual Reality (VR)

A virtual reality headset (Vive, HTC Corporation, New Taipei City, Taiwan) was used to visualize the International Space Station (ISS) mockup. Subjects could: (a) explore the exterior of the ISS (we refer to this as EVA, for extra-vehicular activity) using handrails to ambulate; (b) watch the Earth rotate below; and (c) look at the stars in the sky. Although the subjects’ feet, body weight, and vestibular system were all experiencing 1 G when interacting with the ISS (i.e., ambulating along the handrails), the law of physics for the visual motion were the same as they would be in actual weightlessness. This VR simulation of the ISS while in 1 G condition primed subjects in VR before testing functional task performance with VR + DOS.

The subjects also wore the virtual reality headset while their body was 100% unloaded (simulated 0 G) using ARGOS (Figure 1). Subjects performed various intra-vehicular activities (IVA), such as moving inside the various ISS modules and drilling using a virtual tool controlled by a hand controller. These tasks were designed to encourage use of their hands rather than their feet to move themselves and to perform tasks in a “floating” position, as is the case in a true 0 G environment. VR simulation of the ISS while in 100% unloaded condition primed subjects before making the transition to partial gravity, as would occur in an actual Mars mission landing.

### 2.4. Disorienting Optokinetic Stimulation (DOS)

**Figure 2:**
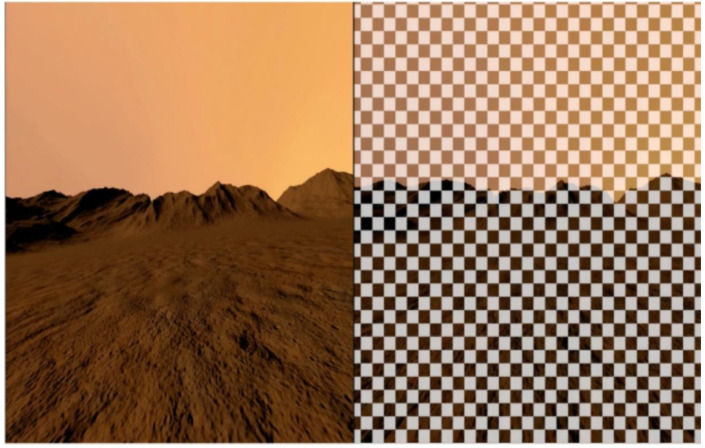
Left: virtual scene of a Martian environment (VR). Right: the virtual Martian scene with the optokinetic stimulation shading layer superimposed (VR + DOS).

A disorienting optokinetic stimulation (DOS) was superimposed onto a scene of the Martian environment (VR + DOS) (Figure 2). The DOS was composed of a checkerboard pattern that moved in roll and yaw in the same direction and velocity as the subject’s head movements. The objective was to mimic the perceptual illusions of a translation of the visual scene reported by astronauts when they tilt their head relative to gravity after spaceflight [3]. When turning the head to the right or left, subjects had the feeling of exaggerated translation to the right or left, respectively.

### 2.5. Tandem Walk

**Figure 3:**
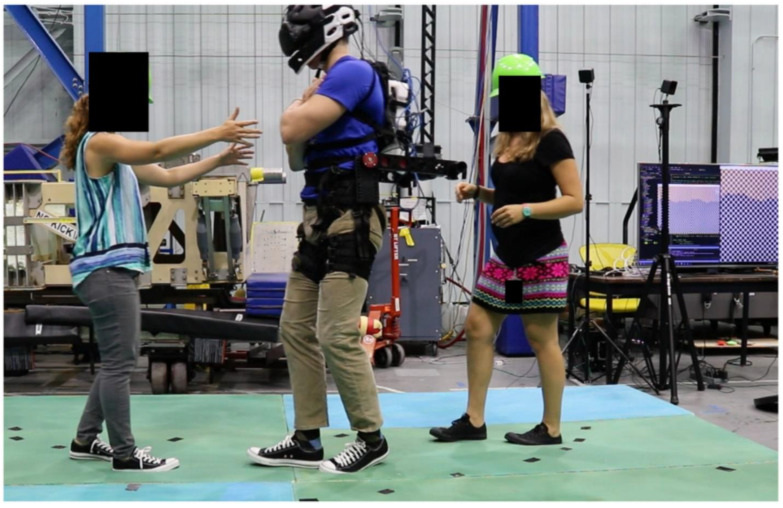
A subject, assisted by 2 operators, is performing the tandem walking test on medium-density foam with 62% of their body weight offloaded (simulated 0.38 G) while visualizing a Martian environment. The subject and operators gave written informed consent for the publication of this image.

Subjects were asked to walk heel-to-toe for 10 steps at a self-selected pace with their arms crossed over their chest. Subjects were then asked to turn 180 degrees and perform another 10 step tandem walk. The laboratory floor was covered by a 10 cm-thick medium density foam (Sunmate Foam, Dynamic Systems, Inc., Leicester, NC, USA) to make proprioceptive information (i.e., ankle proprioception) less reliable when walking, as is the case after adaptation to 0 G [14,15]. During the tandem walking test, subjects wore the virtual reality headset and were attached to the ARGOS suspension system (see below) (Figure 3).

**Figure 4:**
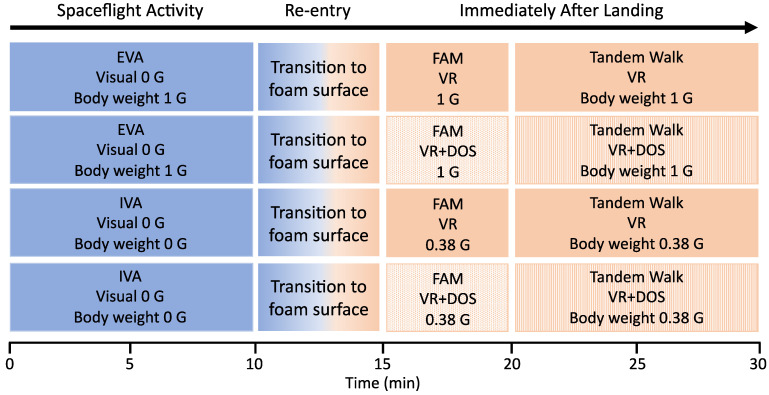
Diagram showing the conditions during the four test sessions. First, the subjects performed extra- or intra-vehicular activities (EVA, IVA) while visualizing the ISS in virtual reality (Visual 0 G) with the body unloaded 0% (1 G) or 100% (0 G). Second, the subjects transitioned to a foam surface, and body unloading simulated the terrestrial (1 G) or Martian (0.38 G) gravitational field. Third, the subjects were familiarized (FAM) with the visual environment and the body unloading conditions. Fourth, the subjects performed the tandem walking test in four conditions: 1 G in VR; 1 G in VR + DOS; 0.38 G in VR; 0.38 G in VR + DOS. The order of sessions was randomized across subjects.

Each subject was tested during four separate sessions that included two spaceflight activities (extra-vehicular activity, EVA; intra-vehicular activity, IVA), two visual conditions (VR, VR + DOS), and three body weights (equivalent to 1 G, 0 G, and 0.38 G) (Figure 4). The protocol for each session was as follows: (a) 10 min of IVA and EVA activities in 1 G and simulated 0 G, respectively, while viewing a virtual mockup of the ISS; (b) a 2 min transition to the foam surface with eyes closed, followed by approximately 3 min to adjust to the virtual scene of Mars and body weight; (c) a 5 min familiarization session where subjects performed a series of tasks while viewing a Martian environment; and (d) the two 20 step tandem walks with VR or with VR + DOS. The test sessions were separated by an average of 4.3 days, and the order of sessions was randomized.

During the IVA activities in simulated 0 G, subjects were able to see, feel, and use handrails in virtual reality to ambulate (Figure 2) and perform a drilling task. These tasks required subjects to become comfortable moving using their hands and become accustomed to working in a “prone” position. The EVA activities included a tour of the ISS in virtual reality and a space walk outside the ISS for watching the Earth rotate below and looking at the stars. During the familiarization session, while viewing the Martian environment in simulated 0.38 G, the tasks included standing from a prone position, a square perimeter walk, vertical jumps, and a small vertical burpee (prone to stand to jump).

### 2.6. Data Analysis

The video of each trial was recorded. Three reviewers examined the videos independently to determine the number of correct steps during each trial. A ‘‘misstep’’ was defined as any of the following: (a) the subject’s stepping foot crossing over the plant foot; (b) the subject stepping to the side before completing the step; (c) the subject’s stepping foot swinging in a wide, arcing path before stepping down; (d) a step duration greater than 3 s; or (e) a gap larger than 10 cm between the heel of the front foot and toe of the back foot when the step was completed [14,15]. Videos for all trials across all sessions for a given subject were pooled, then the order was randomized to minimize reviewer bias based on their awareness of the session. After all reviewers completed their assessments, the median value was used to determine the percent of correct steps for that trial. The percent of correct steps for both trials were then averaged.

One subject in ARGOS was unable to bring their legs close enough together to walk heel-to-toe. The feet of another subject were not visible in the recorded video. Thus, we were unable to assess tandem walk performance for two subjects in 0.38 G and VR. For the remaining eight subjects, repeated-measures analysis of variance (ANOVA) and post hoc *t*-tests were used to compare the percent of correct steps between VR and VR + DOS in 1 G and 0.38 G.

In order to assess the validity of using VR + DOS as an analogue for post flight tandem walk performance, we compared the percent of correct steps during tandem walk on a foam surface in our study with the mean percent of correct steps previously collected during tandem walk on a hard floor with the eyes closed in 13 astronauts after a 6-month stay on board the International Space Station (ISS) [15], in 7 astronauts after a 2-week spaceflight on board the Space Shuttle (STS) [14], and in 10 subjects after a 70-day bed rest in 6-deg head down tilt [15]. For this comparison, a linear effects model was used (fitlm, Statistics Toolbox, Matlab, Mathworks Inc., Natick, MA); the dependent variable was defined as the tandem walk performance in VR + DOS, post bed rest, as well as post Shuttle and ISS missions. Statistical significance was determined using a 0.05 alpha level test.

## 3. Results

None of the subjects experienced stomach discomfort or nausea during the test sessions, although some subjects exhibited other signs of motion sickness, such as sweating, disorientation, and mild pallor. When performing EVA or IVA with 100% of their weight offloaded, many subjects reported slight vertigo and anxiousness about falling. When 62% of their body weight was offloaded, subjects reported increased difficulty performing basic functional tasks, such as walking or maneuvering to a standing position. Some subjects had feelings of disorientation and dizziness that persisted for up to 2 h after they emerged from the VR + DOS condition.

**Figure 5:**
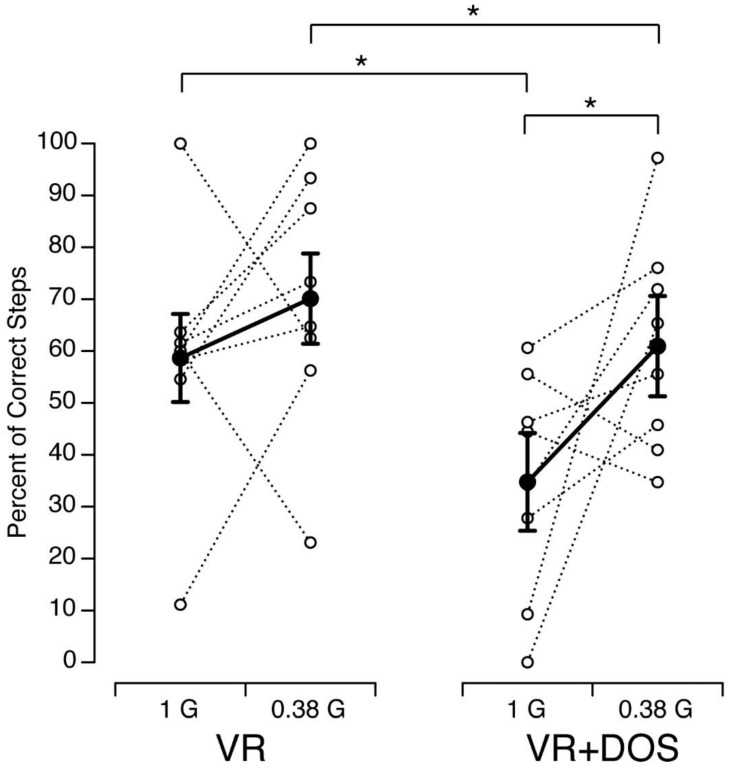
Percent of Correct Steps during the Tandem Walking Test in 8 subjects as a function of gravity level while viewing the virtual Martian scene (VR) or the virtual martial scene with a superimposed optokinetic stimulation (VR + DOS). Open symbols show individual data; filled symbols show mean ± SD. * *p* < 0.05.

The percent of correct steps measured in each subject and condition is shown in Figure 5. Large variations in the percent of correct steps were related to the subjects’ foot size, where they began the tandem walk on the foam surface, and the amount of stumbling during a trial. A repeated-measures ANOVA with two factors (visual conditions: VR, VR + DOS; body weight: 1 G, 0.38 G) indicated that the percent of correct steps was significantly different in the VR and VR + DOS conditions (F(1,31) = 7.62, *p* = 0.01) and was significantly different in 1 G and 0.38 G (F(1,28) = 5.14, *p* = 0.03). However, the interaction between the two factors was not statistically significant (F(1,28) = 0.61, *p* = 0.43). Post hoc paired sample *t*-test indicated that the percent of correct steps was significantly smaller in VR + DOS compared to VR for both gravity levels (offloaded body weights). This decrease was 46.6% in 1 G (*p* = 0.02) and 21.0% in 0.38 G (*p* = 0.02). In both visual conditions, the percent of correct steps increased from 1 G to 0.38 G in six out of eight subjects. This increase was significant in the VR + DOS condition (*p* = 0.04), but not in the VR condition (*p* = 0.18).

**Figure 6:**
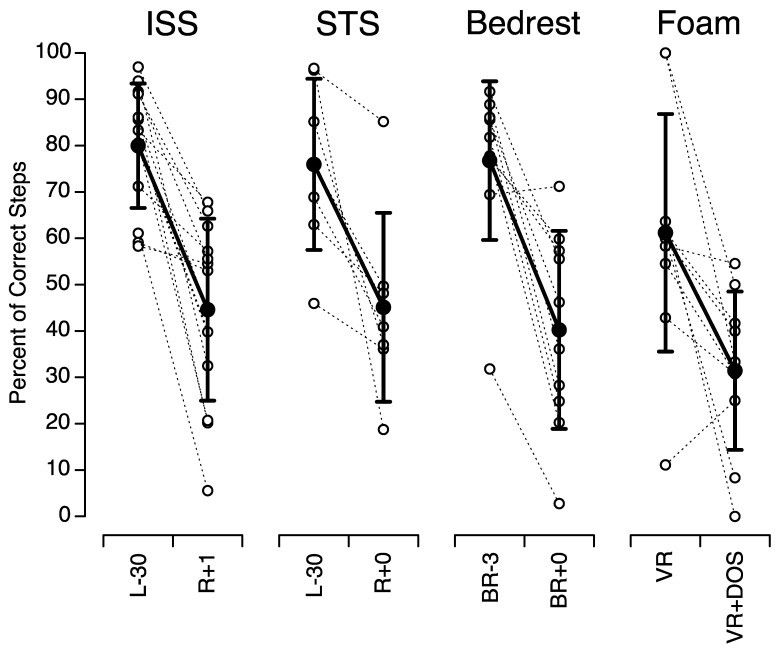
Comparison of the percent of correct steps during tandem walk on a foam surface in our study (Foam) with the mean percent of correct steps during tandem walk on a hard floor with the eyes closed in 13 astronauts before and after a six-month stay on board the International Space Station (ISS) (adapted from Mulavara et al., 2018, Figure 5, p. 1970), in 7 astronauts before and after a 2-week spaceflight on board the Space Shuttle (STS) (adapted from Miller et al., 2018, Figure 3, p. 811), and in 10 subjects before and after a 70-day bed rest in 6 deg head down tilt (adapted from Mulavara et al., 2018, Figure 5, p. 1970). Open symbols show individual data; filled symbols show mean ± SD. L-: day before flight; R+: day after flight; BR-: day before bed rest; BR+: day after bed rest.

Figure 6 compares the percent of correct steps in our study with those measured in astronauts after space missions and bed rest. The mean percent of correct steps during tandem walking on a foam surface in VR + DOS in 1 G in our study (31.4 ± 17.1%; *n* = 10) was not significantly different from the mean percent of correct steps during tandem walking on a hard surface with the eyes closed immediately after a 2-week space flight (45.1 ± 20.4%; *n* = 7, *p* = 0.17) or a 70-day head-down-tilt bed rest (40.2 ± 21.4%; *n* = 10, *p* = 0.32), or 1 day after returning from a 6-month space flight (44.6 ± 19.7%; *n* = 13, *p* = 0.12).

## 4. Discussion

In this study, we investigated how simulated Martian gravity and visual disorientation affect tandem walk performance. Results indicate that tandem walk performance was altered when subjects were viewing a visual disorientation superimposed on a virtual reality scene (VR + DOS). However, in this visual condition, the tandem walk performance was better in 0.38 G than in 1 G. Results also show that the performance of our subjects during the tandem walking test on a foam flooring in VR + DOS was comparable to the performance of astronauts during tandem walk on a hard flooring with eyes closed after spaceflight [14,15].

The purpose of the tandem walking test was to assess changes in dynamic balance control. Dynamic balance during walking is controlled by motor neurons that regulate muscle activity, and by sensory inputs from the vestibular, visual, and somatosensory systems, as well as a central prediction of the afferent signals [23].

### 4.1. Role of the Vestibular System

The tandem walking test is a reliable indicator of vestibular-driven ataxia [24]. Patients with vestibular impairments have impaired tandem walking performance compared to healthy subjects [20]. In addition, it has been shown that tandem walking performance declines with age [25,26]. Unfortunately, the small number of subjects in the present study did not allow us to study the effects of age and gender on the measured responses.

Astronauts returning from spaceflight and walking through an obstacle course on the same compliant foam surface as in our ground-based study exhibited altered locomotor function, with a median 48% increase in the time needed to complete the course [27]. Decrements in computerized dynamic posturography were also seen in the same crewmembers [28]. After a two-week spaceflight, the ability to stand on rails with the feet in tandem (tandem Romberg test) was reduced to 40–90% in Space Shuttle crewmembers [21]. However, a decrease in tandem walk performance, similar to that observed in returning astronauts, was also observed after 60 d and 70 d head-down tilt bed rest in ground-based subjects [14,15,29]. During bed rest and in our study, the vestibular system was still exposed to the 1 G gravitational acceleration; therefore, the decrement in tandem walk performance in all of these studies cannot be solely attributed to decreased vestibular function [15].

### 4.2. Role of the Visual and Somatosensory Systems

Viewing the VR + DOS scene altered the contribution of the vestibulo-ocular reflex for stabilizing gaze. This result is in agreement with computerized dynamic posturography studies showing greater imbalance during optokinetic stimulation [30] and when the visual input is stabilized relative to the head [31] than in darkness. Ground-based studies have also shown that older adults use vision to a greater degree to control head stabilization than do young adults [32,33]. The increased reliance on vision by older adults has been attributed to a decrease in vestibular and proprioceptive function with age [34].

The increased tandem walk performance in ARGOS in 0.38 G could be due to an increase in the role of somatosensory inputs in controlling dynamic balance when the body is unloaded. ARGOS reduces body weight bearing, which modifies the inputs from the receptors in the skin, muscles, bones, joints, and internal organs. Body unloading reduces the inputs from the mechanoreceptors in the soles of the feet. However, other skin areas in contact with the harness provide inputs for the control of dynamic balance. Muscular tone in the leg postural muscles is also reduced due to body unloading, and the efferent signals generated by the motor system are different.

The improved performance of tandem walk when the body weight was unloaded in ARGOS compared to 1 G could be because the body harness in ARGOS helps minimize body sway, thus improving postural stability and gait. The reduced ground reaction force when body weight is unloaded also contributes to an increased cone of stability. The limits of stability are defined by the angles in which the body’s center of gravity moves past the base of support, and when the limits are reached, a correction is required (e.g., a step) to maintain balance. Less ground force reaction allows further deviation from the base of support before a correction is required; thus, less ground force facilitates postural balance and gait. Ground-based studies using an isokinetic testing system have shown that proprioception was significantly correlated with the limits of stability [35].

## 5. Conclusions

Previous investigations by our group showed a significant reduction in the percent of correct steps during the tandem walking test with eyes closed performed by astronauts immediately after a short- or long-duration spaceflight [14,15]. After astronauts return from a spaceflight, the width of their leg placement is exaggerated: they take small steps of irregular length, and they fail to maintain their intended path [4]. Astronauts often experience oscillopsia when they move their head after returning from space, which contributes to disrupted locomotion [2]. The results of the present study indicate that an exaggerated motion of the visual scene during head movements (VR + DOS) in 1 G impairs walking performance on a compliant foam floor in the same manner as in astronauts with eyes closed immediately after spaceflight. The combination of a medium-density foam surface, VR, and optokinetic stimulation could therefore be used as an analog to evaluate the effectiveness of countermeasures for mitigating walking performance after spaceflight. The system could be improved, however, by adding galvanic vestibular stimulation triggered with head movements to alter the contribution of the vestibular inputs, as is the case after spaceflight [36]. Additionally, further work is required to determine if this analog environment could be used to expose crewmembers to the type of visual-vestibular deficits they will experience after landing and determine if preflight or inflight training can improve postflight walking performance [37].

## Data Availability

The data that support the findings of this study are available upon reasonable requests.
